# Mixed-Matrix Membranes Based on Polyetherimide, Metal–Organic Framework and Ionic Liquid: Influence of the Composition and Morphology on Gas Transport Properties

**DOI:** 10.3390/polym14173489

**Published:** 2022-08-25

**Authors:** Sarra Zid, Pierre Alcouffe, Matthieu Zinet, Eliane Espuche

**Affiliations:** Univ Lyon, CNRS, UMR 5223, Ingénierie des Matériaux Polymères, Université Claude Bernard Lyon 1, INSA Lyon, Université Jean Monnet, CEDEX, 69622 Villeurbanne, France

**Keywords:** mixed-matrix membranes, ZIF-8, ionic liquid, morphology, gas transport properties

## Abstract

In this work, membranes based on polyetherimide (PEI), a ZIF-8 metal–organic framework and 1-ethyl-methylimidazolium tetrafluoroborate ionic liquid (IL) were prepared. IL and ZIF-8 contents amounting to 7 wt% and 25 wt%, respectively, were investigated. CO_2_, He and H_2_ transport properties of PEI/IL/ZIF-8 membranes were compared to those obtained for the respective PEI/ZIF-8 and PEI/IL systems. Membranes’ gas permeability and selectivity are discussed as a function of the membrane composition and morphology, and they were assessed in relation to existing experimental and theoretical data from the literature. Promising gas transport properties were obtained using the appropriate combination of ZIF-8 and IL amounts in the PEI matrix. Indeed, an increase in the CO_2_ permeability coefficient by a factor of around 7.5 and the He and H_2_ permeability coefficients by a factor of around 4 was achieved by adding 7 wt% IL and 10 wt% ZIF-8 to the PEI matrix. Moreover, diffusion was evidenced as a governing factor in the studied membrane series.

## 1. Introduction

Global warming is recognized by almost all atmospheric scientists as a significant environmental problem caused by an increase in levels of certain trace gases in Earth’s atmosphere since the beginning of the Industrial Revolution in the mid-18th century. These gases, collectively called greenhouse gases, include carbon dioxide (CO_2_) [1,2,3]. Removing CO_2_ from gas mixtures emitted by human activity is of crucial importance to cutting down its atmospheric concentration increase [4]. Different strategies have been developed for that purpose, including sorbents/solutions methods, cryogenic distillation methods and membrane separation methods [5,6,7,8,9,10,11,12,13]. The latter is a promising technique because of its advantages concerning energy savings, small footprint and environmental sustainability [14,15,16]. More particularly, polymer membranes are the most widely used for separation applications [17,18,19]. However, the challenge of enhancing both gas permeability and selectivity of polymer membranes requires the incorporation of selective and highly permeable fillers into the polymer matrix to form mixed-matrix membranes (MMMs) [20,21,22,23,24,25]. Zeolitic imidazolate frameworks (ZIFs) are a new class of inorganic materials that have been extensively mixed with polymer matrices for gas separation applications in the last decade [26,27,28,29,30,31]. They are characterized by their exceptional thermal and chemical stability [32,33], high sorption capacity and high surface areas and pore volumes [34,35]. Haldoupis et al. [36] showed that these fillers could have important selectivity for CO_2_/CH_4_ mixtures. During the last decade, numerous works have focused on MMMs based on rubbery polymer membranes and zeolitic imidazolate framework (ZIF-8) particles, which have shown a high increase in CO_2_ permeability [37,38,39,40,41]. For example, Nafisi et al. [37] incorporated ZIF-8 within a commercial polyamide-block–ether copolymer (Pebax 2533). The initially good CO_2_ permeability of the semi-crystalline and rubbery matrix was further improved: the permeability increased from 351 to 1287 Barrer for a membrane containing 35 wt% ZIF-8. Xu et al. even showed a higher enhancement in CO_2_ permeation performance by inserting ZIF-8 particles in a Pebax 1657 matrix [38]. The membrane containing 18 wt% ZIF-8 exhibited as high as a 300% increase in CO_2_ permeability in comparison with the neat polymer membrane. Recent work performed on Pebax/ZIF-8 membranes showed the same trends [40,41]. Work was also dedicated to the combination of glassy polymers with ZIF-8. Among the glassy matrices that were used, one can notice Matrimid, (poly-(1,4-phenylene ether-ether-sulfone), poly(2,6-dimethyl-1,4-phenylene oxide) and polyetherimide [29,35,42,43,44,45]. This last amorphous polymer has already been shown to be particularly interesting for membrane applications. It is a thermally stable material from which it is possible to easily form dense or porous membranes [46,47,48,49,50,51]. A few works [29,52,53,54] have aimed at preparing MMMs from polyetherimide (PEI) and ZIF-8, with the aim of improving their gas separation properties. Eiras et al. [52] showed that the addition of ZIF-8 could increase CO_2_ permeability without detrimental effects on CO_2_/CH_4_ and CO_2_/N_2_ selectivities. Moreover, Dai and coworkers [29] reported the development of PEI/ZIF-8 mixed-matrix hollow-fiber membranes, which was dedicated to the improvement in CO_2_/N_2_ selectivity. Zhu et al. [54] also developed hollow-fiber membranes from PEI and low amounts of ZIF-8 (less than 0.08 wt%). A significant increase in O_2_ flux (factor 2.7) and N_2_ flux (factor 3.7) was obtained while the selectivity decreased from 6.9 to 5.0. Some authors showed that the decrease in selectivity could be related to the formation of interfacial defects due to poor adhesion between the particles and the polymer matrix [55]. To face those issues, filler surface modification through alkoxysilane grafting was often performed [56]. Another route could be to add a third component in the medium with the aim of increasing the accessibility to the porous fillers while avoiding defects and maintaining specific interactions towards CO_2_ [57]. Ionic liquids (ILs) are a new alternative for preparing membranes for CO_2_ removal applications [58,59,60]. Their distinct properties, such as negligible vapor pressure and their affinity for capturing CO_2_ molecules, make them very useful today. Blanchard et al. [61] showed that CO_2_ has a very high solubility in 1-Butyl-3-methylimidazolium hexafluorophosphate ([bmim] [PF6]). Other ILs, such as 1-Ethyl-3-methylimidazolium tetrafluoroborate ([Emim] [BF4]), are also recognized for their interesting CO_2_ sorption ability [62]. Moreover, a work performed on a system composed of poly(room-temperature ILs), room-temperature ILs and SAPO-34 particles showed an improvement in the filler–matrix compatibility, as well as an enhancement in the CO_2_ absorption [63]. The same conclusion was reached for Pebax/ZIF-8/IL membranes [57,64]. Although such ternary systems were studied with a Pebax matrix, according to our knowledge, no published work can be found in the literature with a focus on glassy polyetherimide matrices.

In the current work, three-component membranes based on polyetherimide (PEI), ZIF-8 particles and [Emim][BF_4_] ionic liquid were prepared and characterized, and their gas transport properties are reported. The factors governing the gas transport properties in this membrane series were inferred from the comparison of the three-component membranes with the respective two-component systems, e.g., PEI/ZIF-8 and PEI/[Emim][BF_4_] ionic liquid, and the reference PEI membrane.

## 2. Materials and Methods

### 2.1. Materials

**Figure 1:**
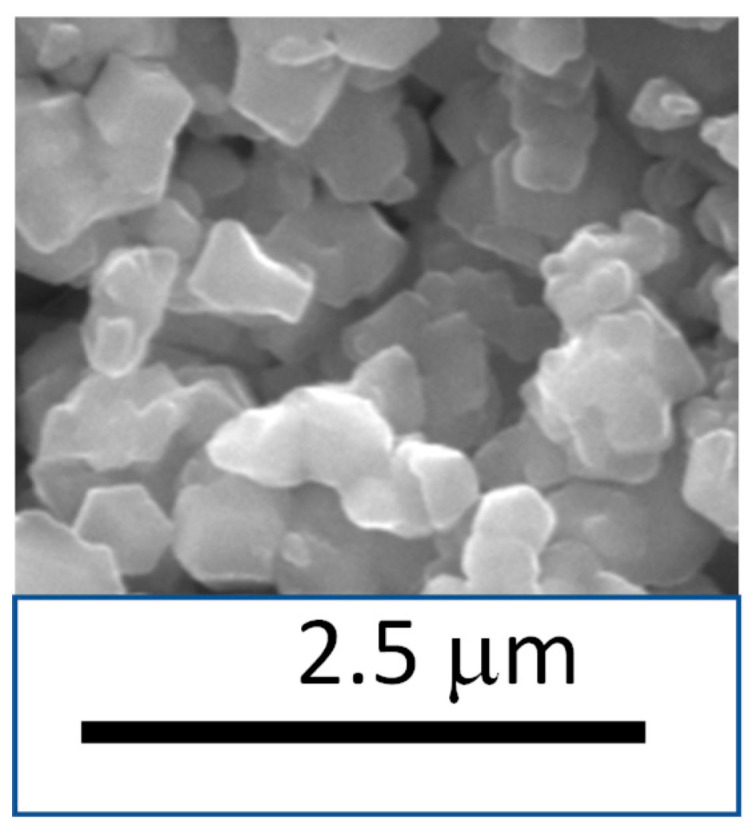
SEM image of ZIF-8 particles.

Table 1 summarizes the chemical structures of the different materials used to prepare the membranes. Polyetherimide granules (PEI, Ultem 1000) with a bulk density of 1.27 g/cm^3^ were purchased from GE Plastics. Basolite Z1200 (ZIF-8) with a bulk density of 0.35 g/cm^3^ and methylene chloride (CH_2_Cl_2_, boiling temperature T_b_ = 40 °C) were purchased from Sigma-Aldrich. 1-ethyl-methylimidazolium tetrafluoroborate ionic liquid (IL, purity > 98%; degradation temperature T_d_ = 450 °C) with a density of 1.387 g/cm^3^ was obtained from Io-Li-Tech. Figure 1 shows that the ZIF-8 nanoparticles exhibited a uniform particle size, which was around 0.7 μm.

### 2.2. Membrane Preparation

Four membrane series were prepared: PEI reference films, PEI films containing an increasing amount of IL (from 2.5 to 20 wt%), mixed-matrix films composed of PEI and ZIF-8 in which ZIF-8 content varied between 10 and 25 wt% and mixed-matrix films combining IL and ZIF-8.

PEI granules were always dried at 120 °C in a vacuum oven for 6 h prior to use.

To obtain the reference PEI film, PEI was dissolved in CH_2_Cl_2_ for almost 1 h at ambient temperature under stirring conditions to prepare the 80 g/L PEI solution. The prepared solution was cast onto a glass plate, and smooth evaporation of the solvent was performed under ambient conditions to obtain membranes with a thickness of around 50 μm.

For PEI/IL films, defined amounts of IL were dissolved in CH_2_Cl_2_. The mixture was mechanically stirred for 30 min. Then, PEI granules (pre-dried) were added to the solution to obtain 80 g/L PEI solution and further stirred for 1 h to ensure complete mixing. The solutions were cast onto glass plates and dried under ambient conditions overnight. The obtained films were named PEI/x IL, where x corresponds to the amount of IL in terms of weight contained in the membranes. xwas kept below 7.5 wt% to keep perfectly stable membranes and thus to avoid any migration of the IL towards the membrane surfaces during membrane storage.

Concerning PEI/ZIF-8 membranes, appropriate amounts of ZIF-8 were dispersed in CH_2_Cl_2_ for 1 h by using a sonication bath. A total of 20 vol% of the PEI solution was added to the ZIF-8 dispersion, and the blend was sonicated for 1 h. In the last step, the rest of the polymer solution was added gradually to the ZIF-8 mixture, and the resulting mixture was kept under mechanical stirring conditions for almost 2 h. The solutions were cast on glass plates and dried at room temperature. The obtained films were denoted by: PEI/y ZIF-8, where y represents the amount of ZIF-8 in terms of weight within the membrane (which increased up to 25 wt%).

The protocol used to prepare the three-component PEI/ZIF-8/IL membranes was as follows: first, the appropriate amount of IL was added to the polymer solution to reach a final IL content of 2.5 or 7 wt% within the membrane. The solution was kept under stirring conditions for almost 1 h. Then, a dispersion of ZIF-8 particles was prepared as described before and sonicated for 1 h. Next, 20 vol% of the polymer/IL solution was added to the ZIF-8 dispersion and kept in the ultrasonic bath for 1 h. Subsequently, the rest of the PEI/IL solution was added to the mixture and sonicated for another 1 h. The obtained solutions were poured onto glass plates and dried under ambient temperature overnight. Two membrane compositions were investigated: PEI/2.5 IL/10 ZIF-8 and PEI/7 IL/10 ZIF-8.

### 2.3. Membrane Characterization

The thermal degradation behavior of the prepared membranes was investigated using the thermo-gravimetric analyzer TGA Q500 (TA Instruments). The mass change versus temperature was measured under air with a heating rate of 10 °C/min over a temperature range of 25–650 °C. Differential scanning calorimeter (DSC) Q200 1854 (TA Instruments) was used to determine the glass transition temperature (Tg) of the polymer matrix by heating samples from 25 to 250 °C at a heating rate of 10 °C/min under a helium atmosphere. Two heating cycles were recorded, and the glass transition temperature Tg values were determined, as usually done for high Tg systems prepared from solvent casting [65], on the thermograms of the first and second cycles using the midpoint method. This allowed us to see, in addition to TGA data, if residual solvent remained in the film. Indeed, in that case, the Tg value of the first scan should be lower than that of the second scan.

The morphology of PEI/IL, PEI/ZIF-8 and PEI/IL/ZIF-8 membranes was observed by scanning electron microscopy (SEM) using a Quanta 250 from FEI. SEM images were taken on the membrane cross section. The samples were prepared by ultra-microtomy at room temperature using a diamond knife to obtain smooth surfaces. They were metallized with carbon. Energy-dispersive X-ray (EDX) analysis was conducted in order to complete SEM observations with information about the chemical composition of the different domains evidenced by SEM.

Water contact angles (θ) were measured with an optical contact angle meter (DSA 100 equipped with CDD2/3 camera, Krüss) via the sessile drop technique. The reported values were the average value obtained from at least six measurements for each sample.

### 2.4. Gas Permeation Analysis

Gas permeation experiments were carried out at 20 °C for He, CO_2_ and H_2_. Three samples were tested for each studied formulation. The permeation cell, consisting of two compartments separated by the membrane, was desorbed under secondary vacuum before each experiment. A 2-bar gas absolute pressure was then applied to the upstream compartment of the cell. The pressure variations in the downstream compartment were measured over time by a datametrics pressure sensor (10 Torr) and allowed us to determine the penetrant flux going through the membrane at any time. The permeability coefficient, *P*, expressed in Barrer units (1 Barrer = 10^−10^ cm_STP_^3^·cm·cm^−2^·s^−1^ cm_Hg_^−1^ = 3.36 × 10^−16^ mol·m·m^−2^·s^−1^·Pa^−1^), was calculated by using the flux value *J* obtained in the steady state:

(1) $$P=\frac{eJ}{A\Delta p}$$

where *A* and *e* are the sample surface area and thickness, respectively, and *Δp* is the trans-membrane pressure. The diffusion coefficient *D* was determined using the time-lag method:

(2) $$D=\frac{e^{2}}{6t_{l}}$$

where *t_l_* is the time lag. According to the solution–diffusion mechanism, the solubility coefficient *S* could be calculated as follows:

*S = P/D* (3)

The membrane’s ideal selectivity values were determined as the ratio of the permeability values for a gas pair (*A*, *B*):

*α_AB_ = P_A_/P_B_* (4)

## 3. Results

In order to better understand the behavior of PEI/IL/ZIF-8 membranes and to determine the factors governing their gas transport properties, it was essential to first analyze the morphology, polymer chain mobility and gas transport properties of the reference binary membranes (PEI/IL and PEI/ZIF-8, respectively). Indeed, depending on its miscibility with the polymer matrix, IL could potentially lead to increased polymer chain mobility and a plasticizing effect, whereas adding filler could impede or favor chain mobility, depending on polymer–filler interactions. Thus, potential opposite trends could be obtained by using both IL and ZIF-8. Moreover, it was also of great interest to see if the morphology of the three-component membranes could be considered as the simple superposition of the morphologies of each binary system or if it could be modified due to favorable interactions between the two added components (IL and ZIF-8).

### 3.1. PEI/IL Membranes

**Figure 2:**
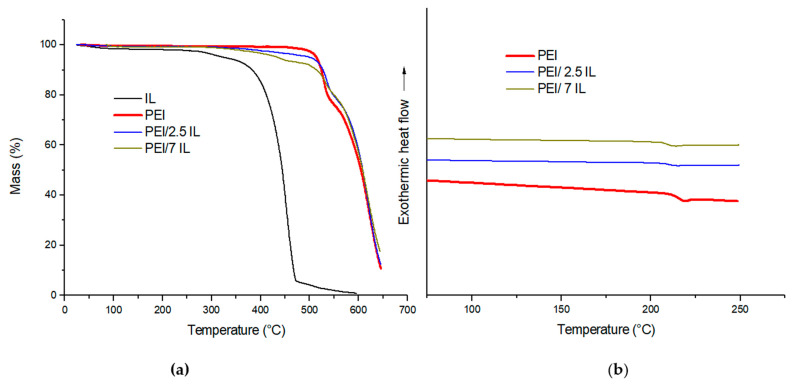
(**a**) TGA mass loss curves and (**b**) DSC thermograms for PEI/x IL (x = 0; 2.5; 7).

The thermal properties of the studied membranes were determined by thermo-gravimetric analysis (TGA) and differential scanning calorimetry (DSC). Figure 2a,b show mass loss curves and representative DSC thermograms for reference PEI membranes and PEI/IL membranes.

It could be observed from Figure 2a that IL degradation began at a lower temperature in comparison with PEI (around 300 °C for IL and above 500 °C for PEI). The small weight loss observed at a low temperature (below 100 °C) for IL could be assigned to the removal of absorbed water. For PEI and PEI/IL membranes, no significant weight loss was observed for the studied membranes below 350 °C, meaning that the membranes did not contain any residual solvent. This was important to check as the presence of residual solvent, even in a low amount, can significantly modify the transport properties of glassy polymers [46]. PEI exhibited a two-stage decomposition (the first one was in the range of 512–550 °C, followed by the second one) as already reported by several authors [66,67,68]. As described in the literature, the first stage corresponded to the decomposition of aliphatic parts through an ether-bond-breaking mechanism, while the second stage could be assigned to the decomposition of aromatic groups involving carboxyl-induced chain breaking. PEI/IL membranes exhibited the first mass loss between 350 °C and 470 °C, which was not observed for neat PEI. It could be observed that this mass loss corresponded to the IL amount introduced within the PEI matrix. At higher temperatures, the mass loss profiles of PEI/IL were similar to that obtained for PEI at a temperature corresponding to the maximum of the derivative mass loss curve (not shown here) equal to 525 °C for the first degradation stage and 620 °C for the second one. From these observations, it could be concluded that IL’s introduction within PEI matrix did not significantly modify the thermal stability of the membranes.

The DSC thermograms of PEI/IL membranes are presented in Figure 2b. It is noteworthy that the *T_g_* values of all membranes were identical between the first and second scan, confirming that the membranes did not contain residual solvent. The PEI reference membrane exhibited a glass transition temperature of 216 °C, which is in good agreement with *T_g_* values reported in the literature [52]. The glass transition temperature slightly shifted to lower values when IL was added to the polymer matrix (*T_g_*_PEI/2.5 IL_ and *T_g_*_PEI/7 IL_ values are around 211 °C). Since *T_g_* value of the IL is around −99 °C [69], the decrease in *T_g_* value observed for PEI/IL membranes could be assigned to a miscibility phenomenon between the ionic liquid and the polymer matrix. The Fox law (Equation (5)) was used in order to determine the amount of IL dissolved within the PEI matrix:

(5) $$\frac{1}{T_{g}}=\frac{w_{PEI}}{T_{gPEI}}+\frac{w_{IL}}{T_{gIL}}$$

where $w_{PEI}$ and *w_IL_* are the weight fractions of PEI and IL in the blend, and *T_g_*, *Tg_PEI_* and *Tg_IL_* are the glass transition temperatures of the blend, PEI and IL, respectively.

The content of IL dissolved in the PEI matrix was found to be 0.57 wt% for both PEI/2.5IL and PEI/7IL, which is far from the amount of IL that was mixed with the PEI matrix. Thus, a low miscibility degree between the PEI matrix and the ionic liquid was obtained in our membranes.

**Figure 3:**
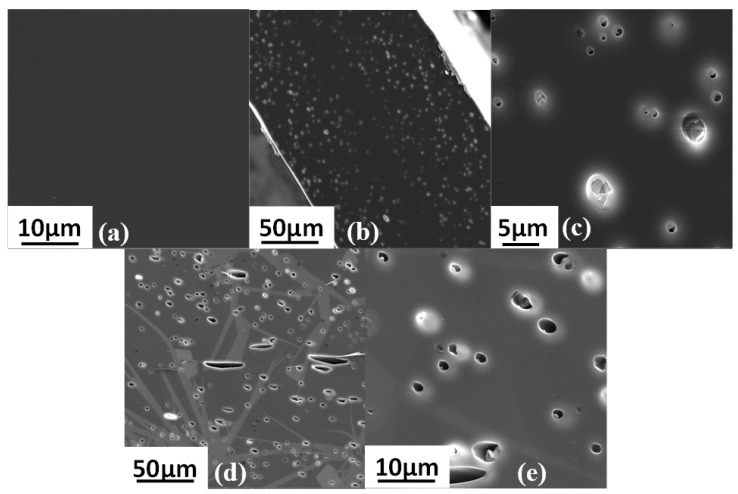
SEM images for the cross section morphologies of (**a**) neat PEI, (**b**,**c**) PEI/2.5 IL membranes and (**d**,**e**) PEI/7 IL membranes.

Cross-sectional SEM images of neat PEI, PEI/2.5 IL and PEI/7 IL membranes are presented in Figure 3a–e.

**Figure 4:**
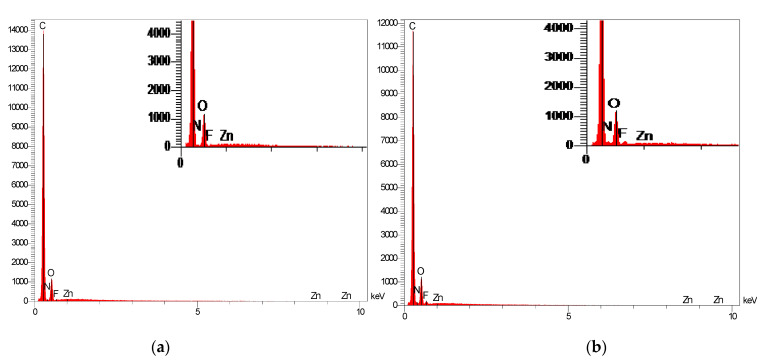
EDX spectra performed (**a**) on the continuous matrix phase and (**b**) on the dispersed domains.

Figure 3a reveals the homogenous smooth cross section for the neat PEI membrane. Introducing ionic liquid in the polymer matrix led to the formation of micrometer size domains that were uniformly dispersed in the polymer matrix (Figure 3b–e). The EDX spectra performed on the continuous matrix phase and on the dispersed domains, respectively, confirmed that the dispersed domains were composed of IL. Indeed, the presence of a small signal relative to Fluorine that could be assigned to IL was observed in the EDX spectrum of the dispersed domains (Figure 4b). This signal was logically not observed for the continuous matrix composed of PEI (Figure 4a). Thus, the phase separation observed for PEI/IL systems was in total agreement with the low miscibility degree between IL and PEI evidenced by DSC analyses.

From SEM images, it could also be observed that part of the IL domains was also located near to the film surfaces, as shown in Figure 3b. Moreover, the more the IL content increased, the more numerous and the larger the dispersed domains corresponding to IL were.

The water contact angle values measured for the membranes decreased from 88.8 ± 3.0° to 87.0 ± 1.8° and 83.2 ± 2.1° as the IL amount increased from 0 to 2.5 and 7 wt%. This result reflects the impact of the higher polarity of the IL with respect to the PEI matrix and location of part of the IL domains near the film surfaces, as already evidenced through SEM analyses.

The gas transport properties of the membranes were determined, and the results are summarized in Table 2.

Whatever the gases, the permeability coefficients of PEI/IL remained close to those obtained for neat PEI. This behavior is in agreement with the trends already observed for other polymer/IL systems that contain low IL amounts [62,70]. For our membranes, as IL formed dispersed domains and was introduced in a relatively low amount, it could be assumed that the gas transport properties were mainly governed by the continuous PEI matrix.

### 3.2. Polymer/ZIF-8 Membranes

**Figure 5:**
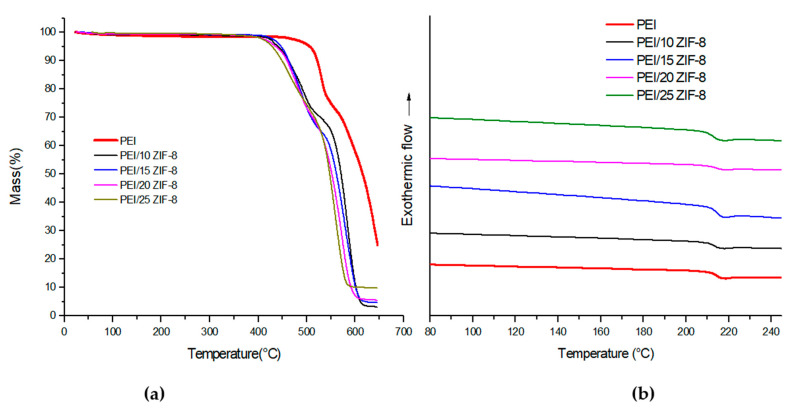
(**a**) TGA mass loss curves and (**b**) DSC thermograms of the mixed-matrix membranes.

The mass loss curves of the PEI/ZIF-8 membranes, for ZIF-8 amounts ranging between 10 and 25 wt%, were compared to that of neat PEI in the temperature range of 25–650 °C (Figure 5a)

No significant mass loss was observed at temperatures below 400 °C, showing that no residual solvent was present in the membranes. Although all mass loss curves exhibited the same global shape, some differences could be observed as a function of the membrane composition: the mass loss curves shifted towards lower temperatures after the introduction of ZIF-8 fillers within the PEI matrix, and the shift slightly increased as the ZIF-8 content increased within the matrix. It could also be observed that the first mass loss (observed in the temperature range of 400–520 °C) increased as the ZIF-8 amount increased, probably due to the collapse of the metal–organic framework in that range of temperature. Indeed, the decomposition of ZIF-8 under air was reported to take place in the temperature range of 375–500 °C and to lead to 35% residue [71]. As expected, the value of the residue at high temperatures observed for our membranes increased as the filler content increased. It is noteworthy that all PEI/ZIF-8 samples remained thermally stable up to 420 °C.

The values of the glass transition temperature of the membranes were similar at the first and second DSC scan, confirming, in agreement with TGA data, the absence of residual solvent in our membranes. It can be seen from Figure 5b that the glass transition temperature values of the mixed-matrix membranes did not significantly change with respect to that of neat PEI. The *T_g_* value decreased from 216 to 212 °C going from 0 to 25 wt% ZIF-8. An increase in the *T_g_* value is usually associated with strong filler–matrix interactions [35]. It could then be concluded that in our membranes, neither strong filler–matrix interactions nor polymer chain mobility impediment occurred.

**Figure 6:**
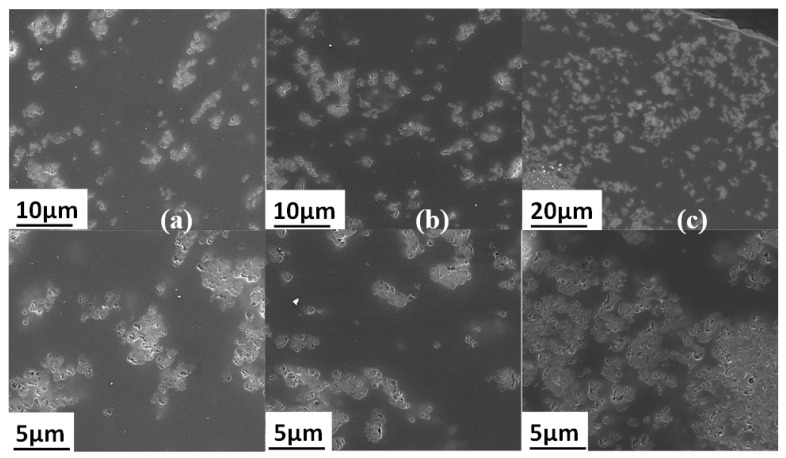
SEM images of (**a**) PEI/10 wt% ZIF-8 (**b**) PEI/15 wt% ZIF-8 and (**c**) PEI/20 wt% ZIF-8.

SEM analysis was performed to investigate the filler dispersion within the PEI matrix. The images of the membranes’ cross sections are provided in Figure 6.

**Figure 7:**
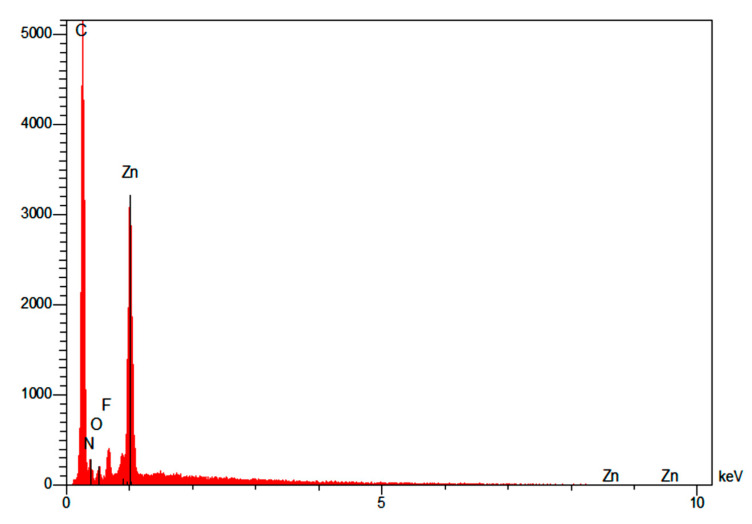
EDX spectra performed on the small aggregates observed in PEI/10 wt% ZIF-8.

The SEM images show that ZIF-8 particles were homogenously dispersed within the polymer matrix as small aggregates a few micrometers in size. Additionally, the EDX analysis performed on the dispersed domains (Figure 7) clearly showed the presence of an intense peak characteristic of Zn species in agreement with the chemical composition of the fillers. It is noteworthy that for PEI/20 wt%ZIF-8, some ZIF-8 aggregates started to percolate through the thickness of the membranes.

The water contact angle values measured for the membranes decreased from around 10° going from neat PEI (θ = 88.8 ± 3.0°) to PEI/ZIF-8 (78.8 ± 4.7°, 79.1 ± 3.5°, 77.2 ± 2.9°, 79.0 ± 3.7° for ZIF-8 amounts of 10, 15, 20 and 25 wt%, respectively). This trend could be assigned to the presence of ZIF-8 nanoparticles near to the film surfaces (as can be seen in the upper right of Figure 6c, where a film surface can be distinguished) independently of the ZIF-8 amount.

Table 3 presents the permeability coefficients measured for He, H_2_ and CO_2_ as well as the CO_2_ diffusion and solubility coefficients. The H_2_/CO_2_ selectivity values are also reported in Table 3.

As the ZIF-8 loading increased up to 25 wt%, the permeability to all gases increased to four times the neat PEI membrane’s value. Moreover, it could be observed that the evolution of the CO_2_ diffusion coefficients followed the same trend as that observed for the permeability, whereas the solubility coefficient was not significantly modified by the presence of fillers. Thus, diffusion could be considered as the governing factor in this membrane series.

**Figure 8:**
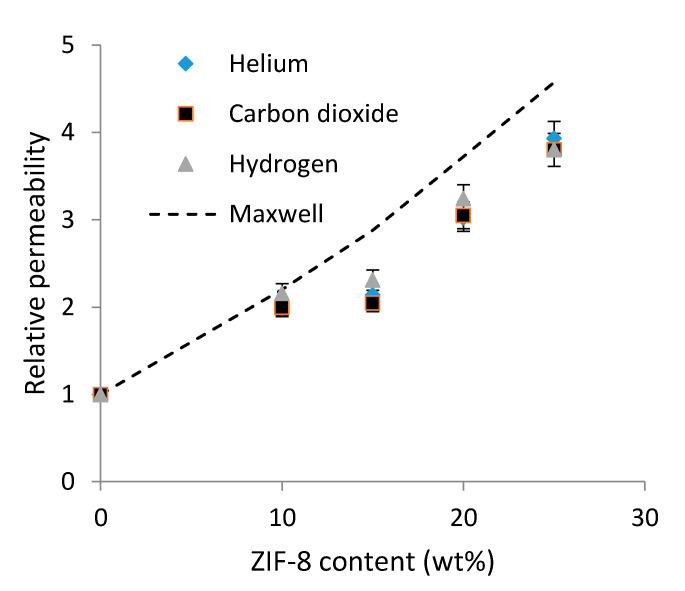
Relative permeability variation as a function of ZIF-8 content for the three studied gases: comparison with theoretical data obtained from Maxwell law.

The relative permeability, defined as the permeability of the PEI/ZIF-8 membranes ratioed by the permeability of the neat PEI, was calculated for each gas, and the obtained values are reported in Figure 8. Moreover, the theoretical relative permeability values were calculated for CO_2_ using the Maxwell model and are reported by the dotted line in Figure 8. The Maxwell model is often used to predict the gas permeation behavior of mixed-matrix membranes containing spherical fillers [55,72]. The Maxwell equation can be written as follows:

(6) $$P=P_{PEI}\frac{\left(P_{ZIF8}+2P_{PEI}-2f\left(P_{PEI}-P_{ZIF8}\right)\right)}{\left(P_{ZIF8}+2P_{PEI}+f\left(P_{PEI}-P_{ZIF8}\right)\right)}$$

where *f* is the filler volume fraction, *P_PEI_* is the permeability of neat PEI, and *P_ZIF_*_8_ is the permeability of the fillers. The CO_2_ permeability of the fillers was considered to be equal to 3300 Barrer as reported by Xu et al. [38]. It should be noted that other values of ZIF-8 permeability could be found in the literature (2918. 6 Barrer in [73]). However, they remained in the same range, and it was ensured that the small deviation observed between them (around 10%) had no significant impact on the calculated values of the permeability.

One could observe from Figure 8 that the relative permeability values did not depend on the gas nature and increased as the ZIF-8 amount increased. This result underlined that adding ZIF-8 allowed for the formation of new gas transport paths inside the material. However, ZIF-8 did not act as selective porous particles for the considered gases (He, H_2_ and CO_2_).

It could also be noticed that the CO_2_ experimental data were in good agreement with the theoretical ones for PEI/10 ZIF-8. For higher ZIF-8 amounts, the experimental data layed below the theoretical curve, and the difference was around 20%. Maxwell law considers ideal binary systems in which each component keeps its initial properties. It could then be suspected that when the ZIF-8 fillers were embedded at a high content in the polymer matrix, their porous structure was not totally available for gas diffusion.

**Figure 9:**
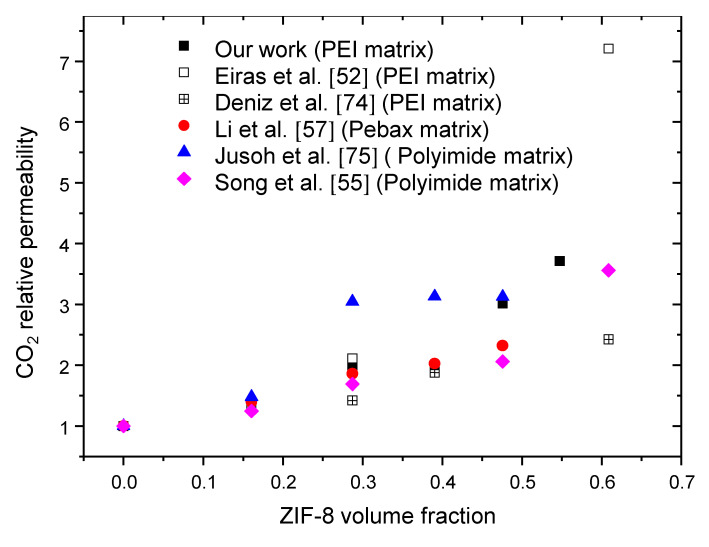
Comparison of our experimental CO_2_ relative permeability with data from the literature obtained for several systems based on ZIF-8 and different polymer matrices [52,55,57,74,75].

Finally, the CO_2_ relative permeability values obtained for our systems were compared to several literature data relative to mixed-matrix membranes containing ZIF-8 particles (Figure 9).

For all systems, the evolution of CO_2_ relative permeability as a function of the filler volume fraction exhibited an increasing trend. It is noteworthy that the CO_2_ relative permeability values obtained in this work were slightly higher than those reported in the literature for similar systems [52,75]. Moreover, considering an extended range of mixed-matrix membranes based on ZIF-8, we could conclude from Figure 9 that the performances of our membranes were promising.

Thus, although the gas selectivity value of the PEI/ZIF-8 membranes remained close to that of the reference PEI, the increase in permeability with the filler amount without any impact on the gas selectivity was a beneficial feature for gas separation application. It could be noticed that the same trend was observed by Eiras et al. [52] for CO_2_/N_2_ gas separation considering PEI/ZIF-8 systems and Li et al. [57] for CO_2_/N_2_ separation considering the Pebax/ZIF-8 system.

### 3.3. PEI/IL/ZIF-8 Membranes

The aim of this part was to investigate whether a synergistic effect could be obtained by using both IL and ZIF-8 at low IL and ZIF-8 contents within the PEI matrix. To achieve that goal, the ZIF-8 particle amount was fixed at 10 wt%, and two IL contents, 2.5 wt% and 7 wt%, respectively, were considered.

**Figure 10:**
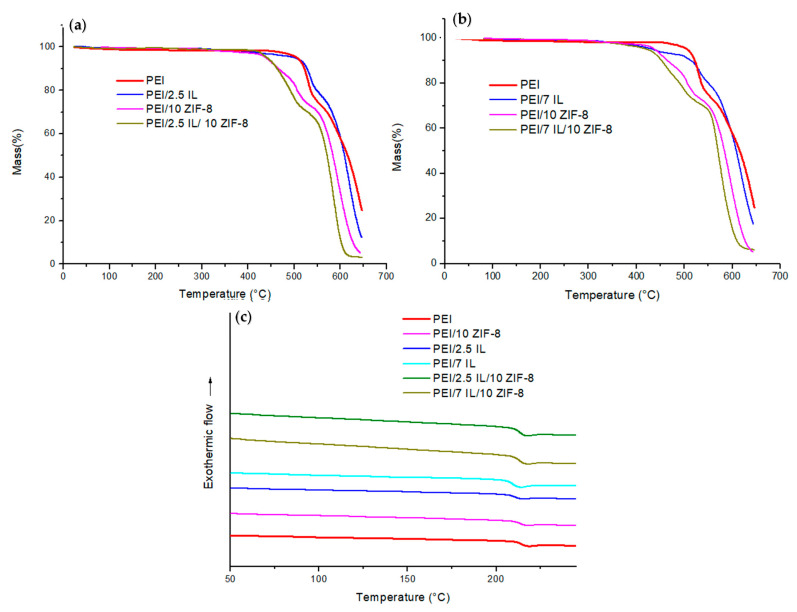
(**a**,**b**) TGA and (**c**) DSC plots of the studied membranes.

Figure 10 compares the TGA and DSC plots of the three-component membranes with those of the corresponding two-component membranes. The thermograms of the neat PEI film have also been included in the figures as references.

The TGA results reported in Figure 10a,b evidence that, in the studied range of compositions, the thermal degradation curves of the three-component PEI/IL/ZIF-8 membranes were close to those of the corresponding PEI/ZIF-8 systems, whereas neat PEI and PEI/IL membranes exhibited higher degradation temperatures. It could then be concluded that ZIF-8 played a major role in the membrane degradation curve independently of the presence or absence of IL in the system. The DSC thermograms (Figure 10c) showed that the *T_g_* of the three-component membranes was around 213 °C, which is similar to that measured for the two-component PEI/ZIF-8 (212 °C) and PEI/IL systems (211 °C). All synthesized membranes thus exhibited good thermal stability and glassy behavior over an extended range of temperature.

**Figure 11:**
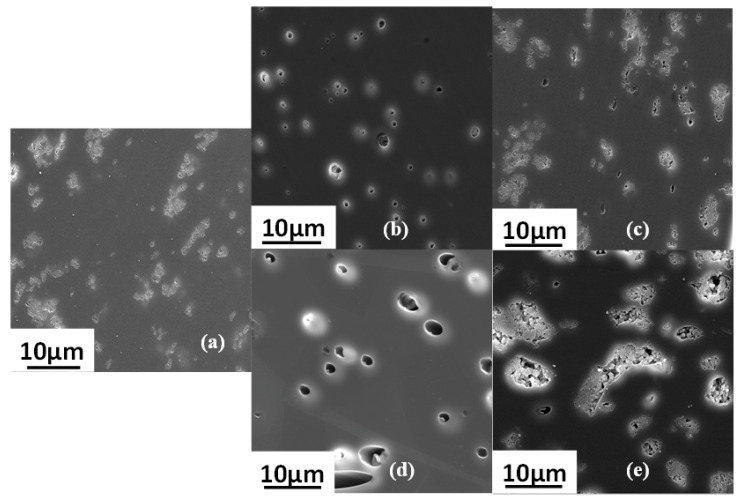
SEM images of the membranes cross sections: (**a**) PEI/10 ZIF-8; (**b**) PEI/2.5 IL; (**c**) PEI/2.5 IL/10 ZIF-8; (**d**) PEI/7 IL; and (**e**) PEI/7 IL/10 ZIF-8.

SEM images of the cross sections of the three-component mixed-matrix membranes and of the corresponding two-component systems are presented in Figure 11.

Surprisingly, IL domains could not be distinguished from the SEM images of PEI/IL/ZIF-8 membranes (Figure 11c,e). Moreover, by comparing the SEM images of PEI/ZIF-8 (Figure 11a) and PEI/IL/ZIF-8 (Figure 11c,e), it could be observed that ZIF-8 aggregates were less compact when the membrane contained IL. It seemed difficult to assert that the IL was dissolved in the PEI matrix in view of the results previously discussed concerning the PEI/IL systems. One might then assume that IL was located in the domains including the ZIF-8 fillers, which would explain why these aggregates were less compact. EDX analyses were then performed in three different locations to confirm the presence of IL in the domains containing the fillers.

**Figure 12:**
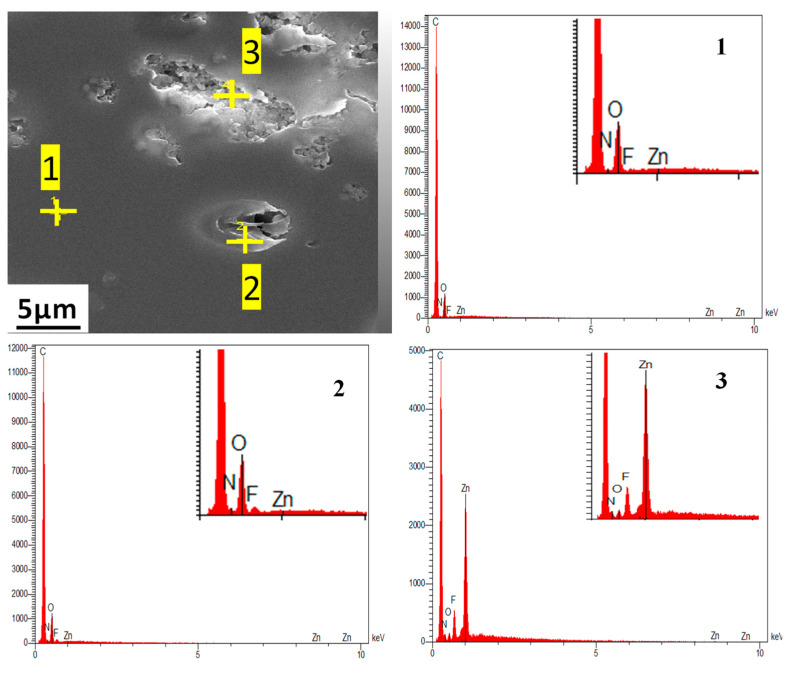
EDX spectra characterizing the different domains observed in SEM image of PEI/7 IL/10 ZIF-8.

It can be seen in Figure 12 that, at point 1 in the SEM image, located in the continuous matrix, no characteristic signature of IL was found, confirming the absence of IL in the polymer matrix. At point 2, located in the external part of a dispersed domain comprising ZIF-8, a small peak related to the presence of F species was detected, evidencing the presence of IL around ZIF-8 particles. Moreover, at point 3, IL and ZIF-8 signatures (the F peak and Zn peak, respectively) were observed, confirming the presence of both ZIF-8 and IL in the small aggregates. It could be concluded that the dispersed domains observed in the continuous PEI matrix were composed of both ZIF-8 and IL resulting from a higher ZIF-8/IL affinity compared to IL/PEI affinity.

The water contact angle values measured for the surface of the three-component membranes were similar (79.0 ± 4.5° and 78.8 ± 3.8° for ° PEI/2.5 IL/10 ZIF-8 and PEI/7 IL/10 ZIF-8). These values are close to the values measured for the PEI/10 ZIF-8 membrane (78.8 ± 4.7°), underlining the presence of ZIF-8 particles near to the film surfaces.

The effect of both IL and ZIF-8 on the membranes’ gas transport properties was investigated. Such a combination was studied for a Pebax matrix in the literature [57,64]. A significant increase in both CO_2_ permeability and CO_2_/N_2_ selectivity was achieved by adding moderate amounts (around 10–15%) of both ZIF and IL in the rubbery Pebax matrix. This behavior was assigned to an increase in the filler–matrix compatibility by adding IL. According to our knowledge, no study has been performed on the glassy polyetherimide/ZIF-8/1-ethyl-methylimidazolium tetrafluoroborate ionic liquid.

The values of the gas transport properties measured for our membranes are presented in Table 4.

Comparing the permeability values of the PEI/IL/ZIF-8 membranes to those of PEI/IL and PEI/ZIF-8 membranes (Table 2 and Table 3, respectively), one could determine the drastic increase in gas flux by adding both IL and ZIF-8 in the PEI matrix, especially for the PEI/7 IL/10 ZIF-8 membrane. Combining 7 wt% IL with 10 wt% ZIF-8 also allowed for a significant increase in the CO_2_ diffusion coefficient with respect to the PEI/10 ZIF-8 membrane (by a factor of around 10) and neat PEI and PEI/7IL (by a factor of around 15). The CO_2_ solubility coefficient only slightly varied (decreasing by a factor of around 2 with respect to neat PEI and PEI/10 ZIF-8 membranes), which confirmed the prevailing role of the diffusion coefficient in the gas transport mechanism. The introduction of IL in a high enough amount allowed for obtaining less-dense ZIF-8 aggregates where ZIF-8 particles were surrounded by IL species, as shown by the SEM images in Figure 11 (going from Figure 11a,c,e) and related EDX analyses. Due to their low *T_g_* value, IL species allowed for the formation of an interfacial area between ZIF-8 and the matrix with a high mobility, promoting gas diffusion and better accessibility to ZIF-8 porous structure in comparison with PEI/10 ZIF-8 membranes, for which the porous fillers were surrounded by glassy polymer chains. In particular, the observed decrease in *α_H2/CO2_* values by a factor of around 2 when comparing PEI/IL/ZIF-8 membranes with neat PEI and binary PEI/IL and PEI/ZIF-8 membranes underlined that the diffusion of the larger CO_2_ gas molecules was promoted in comparison with He and H_2_, which had lower kinetic diameters. It could then be concluded that larger free volumes, most probablylocated in ZIF-8 particles became available for CO_2_ diffusion in the PEI/IL/ZIF-8 membrane.

**Figure 13:**
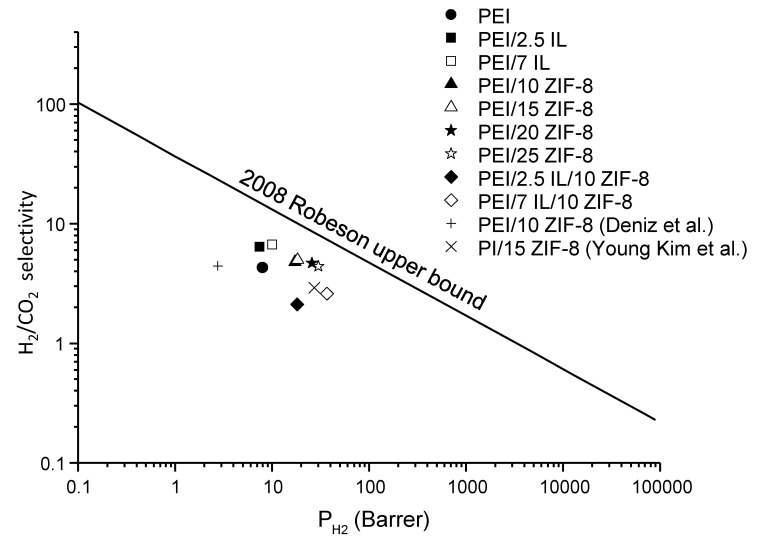
H_2_/CO_2_ selectivity of the prepared membranes versus H_2_ permeability, compared to literature data [75,77].

Finally, in order to have an overview of the performances of the three membranes series synthesized in this work, a plot of their H_2_/CO_2_ selectivity versus H_2_ permeability is presented in Figure 13. The data are compared to the upper-bound line determined by Robeson [76] as well as to results reported for PEI/ZIF-8 systems in the literature [75,77].

Our two-component and three-component membranes exhibited a significant increase in permeability with respect to neat PEI with experimental points lying near the upper-bound line. It is noteworthy that the point relative to PEI/7 IL/10 ZIF-8 was located in the vicinity of those relative to PEI/20 ZIF-8 and PEI/25 ZIF-8, showing the possibility of reaching interesting membranes performances while reducing the filler amounts within the membranes.

## 4. Conclusions

In this study, a step-by-step methodology was used to develop and characterize series of PEI-based membranes with enhanced gas transport properties. It was shown that IL incorporation at a low content in PEI matrices did not lead to significant modification of gas permeability. On the other hand, the addition of ZIF-8 particles to PEI membranes was a good alternative for increasing the permeability, with a main impact on gas diffusion. It was underlined that when the ZIF-8 fillers were embedded at a high content in the polymer matrix, their porous structure was not totally available for gas diffusion. Then, three-component systems (PEI/IL/ZIF-8) were developed. In these membranes, IL was located around the fillers and allowed for the formation of less-compact filler aggregates. Moreover, IL species forming an interfacial area with a high mobility between ZIF-8 and the matrix could promote gas diffusion as well as better accessibility to the ZIF-8 porous structure, especially for CO_2_. All prepared PEI/ZIF-8 and PEI/IL/ZIF-8 membranes had interesting permeability and H_2_/CO_2_ separation properties with experimental points lying near the Robeson upper-bound line. More particularly, the membrane based on PEI, 7 wt% IL and 10 wt% ZIF-8 exhibited properties close to those obtained with membranes based on PEI and 20 wt% or 25 wt% ZIF-8, showing the possibility of reaching high-performance membranes while keeping low filler amounts.

## Figures and Tables

**Table 1:** Chemical structure of the base materials used for two-component and three-component membranes.

Name	Abbreviation	Chemical Structure
Ultem 1000	PEI	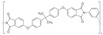
Basolite Z1200	ZIF-8	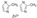
1-Ethyl-3-methyl imidazolium Tetrafluoroborate	[Emim][BF_4_]	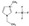

**Table 2:** Gas permeability coefficients of PEI/IL membranes (the uncertainty was better than 5% for *P*).

	*P_He_* (Barrer)	*P_CO2_* (Barrer)	*P_H2_* (Barrer)
PEI	9.2	1.8	7.9
PEI/2.5 IL	8.6	1.2	7.4
PEI/7 IL	9.1	1.5	8.1

**Table 3:** Gas transport properties of the PEI/ZIF-8 membranes (the uncertainty was better than 5% for *P* and *D* and better than 10% for *S* and *α*).

	*P_He_* (Barrer)	*P_CO2_* (Barrer)	*P_H2_* (Barrer)	*D_CO2_* ×10^−9^ (cm^2^/s)	*S_CO2_* (cc_STP_·cm/cm_Hg_)	*α_H2/CO2_*
PEI	9.2	1.8	7.9	1.6	0.11	4.4
PEI/10 ZIF-8	18.3	3.6	17.1	2.7	0.13	4.8
PEI/15 ZIF-8	19.7	3.7	18.3	2.9	0.12	5.0
PEI/20 ZIF-8	27.8	5.5	25.6	4.8	0.11	4.7
PEI/25 ZIF-8	36.2	6.8	30.0	6.7	0.10	4.4

**Table 4:** Gas permeability, diffusivity and selectivity of the prepared membranes (the uncertainty was better than 5% for *P* and *D* and better than 10% for *S* and *α*).

	*P_He_* (Barrer)	*P_CO2_* (Barrer)	*P_H2_* (Barrer)	*D_CO2_* ×10^−9^ (cm^2^/s)	*S_CO2_* (cc_STP_·cm/cm_Hg_)	*α_H2/CO2_*
PEI/2.5 IL/10 ZIF-8	20	8.5	18	6.1	0.14	2.1
PEI/7 IL/10 ZIF-8	28.3	14	36.6	25	0.056	2.6

## Data Availability

Data will be made available on request.
